# Self-guided digital acceptance and commitment therapy for fibromyalgia management: results of a randomized, active-controlled, phase II pilot clinical trial

**DOI:** 10.1007/s10865-023-00429-3

**Published:** 2023-06-29

**Authors:** Stephanie Catella, R. Michael Gendreau, Allison C. Kraus, Nicolette Vega, Michael J. Rosenbluth, Sherry Soefje, Shishuka Malhotra, Juan V. Luciano, Lance M. McCracken, David A. Williams, Lesley M. Arnold

**Affiliations:** 1 Swing Therapeutics, Inc, San Francisco, CA USA; 2 Gendreau Consulting, LLC, Poway, CA USA; 3 Excell Research, Inc, Oceanside, CA USA; 4 Neuro-Behavioral Clinical Research, Inc, North Canton, OH USA; 5 Department of Clinical and Health Psychology, Universitat Autónoma de Barcelona, Barcelona, Spain; 6 Teaching, Research & Innovation Unit, Parc Santari Sant Joan de Déu, St. Boi de Llobregat, Spain; 7 Department of Psychology, Uppsala University, Uppsala, Sweden; 8 Chronic Pain and Fatigue Research Center, Department of Anesthesiology, University of Michigan, Ann Arbor, MI USA; 9 Department of Psychiatry and Behavioral Neuroscience, University of Cincinnati, Cincinnati, OH USA

**Keywords:** Acceptance and Commitment Therapy, Digital ACT, Fibromyalgia, Chronic pain, Prescription digital therapeutic, Smartphone-delivered ACT, Smartphone-delivered CBT

## Abstract

Although empirically validated for fibromyalgia (FM), cognitive and behavioral therapies, including Acceptance and Commitment Therapy (ACT), are inaccessible to many patients. A self-guided, smartphone-based ACT program would significantly improve accessibility. The SMART-FM study assessed the feasibility of conducting a predominantly virtual clinical trial in an FM population in addition to evaluating preliminary evidence for the safety and efficacy of a digital ACT program for FM (FM-ACT). Sixty-seven patients with FM were randomized to 12 weeks of FM-ACT (n = 39) or digital symptom tracking (FM-ST; n = 28). The study population was 98.5% female, with an average age of 53 years and an average baseline FM symptom severity score of 8 out of 11. Endpoints included the Fibromyalgia Impact Questionnaire-Revised (FIQ-R) and the Patient Global Impression of Change (PGIC). The between-arm effect size for the change from baseline to Week 12 in FIQ-R total scores was *d* = 0.44 (least-squares mean difference, − 5.7; SE, 3.16; 95% CI, − 11.9 to 0.6; *P* = .074). At Week 12, 73.0% of FM-ACT participants reported improvement on the PGIC versus 22.2% of FM-ST participants (*P* < .001). FM-ACT demonstrated improved outcomes compared to FM-ST, with high engagement and low attrition in both arms. Retrospectively registered at ClinicalTrials.gov (NCT05005351) on August 13, 2021.

## Introduction

Fibromyalgia (FM) is one of the most common chronic widespread pain disorders, estimated to affect 1.75−6.4% of the U.S. population (Vincent et al., 2013; Walitt et al., 2015). Genetics, infections, trauma, and emotional distress have all been implicated as contributing factors in the development of FM (Clauw, 2014). FM is considered a centralized pain state; the musculoskeletal pain of FM is often accompanied by fatigue, cognitive issues including problems with attention and memory, sleep disturbances, mood and anxiety symptoms, and other somatic symptoms (Clauw, 2014). Due to the complexity and heterogeneity of FM symptoms, the best therapeutic outcomes are typically achieved with a multimodal approach that integrates both pharmacological and nonpharmacological interventions (Clauw, 2014; Hassett & Gevirtz, 2009; Macfarlane et al., 2017; Sarzi-Puttini et al., 2008). Cognitive behavioral therapy (CBT) is considered a first-line treatment with demonstrated level 1 A evidence for both efficacy and tolerability in FM management (Ehde et al., 2014; Kollner et al., 2012) and is recommended by international guidelines (Buckhardt et al., 2005; Fitzcharles et al., 2013; Hauser et al., 2008; Macfarlane et al., 2017).

Acceptance and Commitment Therapy (ACT), a behavioral therapy under the umbrella of CBT, has been empirically validated for the management of FM and other chronic pain conditions in internet-delivered, one-on-one, and group settings (Bernardy et al., 2019; Du et al., 2021; Gandy et al., 2022; Hayes et al., 1999; Herbert et al., 2022; Lin et al., 2017; Luciano et al., 2014; Morcillo-Munoz et al., 2022; Rickardsson et al., 2021; Simister et al., 2018; Steiner et al., 2013; Trindade et al., 2021; Wicksell et al., 2013). As a third wave behavioral therapy, ACT aims to promote healthy psychological and behavioral processes rather than encourage patients focus on reducing or eliminating physical and emotional symptoms. Specifically, ACT aims to prioritize the promotion of psychological flexibility processes including acceptance, defusion, mindfulness, self-as-context, values, and commitment to values-based actions. In the context of FM and other chronic pain conditions, ACT teaches acceptance and mindfulness strategies for uncontrollable symptoms, together with values-based commitment and behavior change strategies to prevent symptom flares, increase quality of life, and improve physical and emotional functioning (McCracken & Morley, 2014). ACT guides individuals to change their expectations from the elimination of pain to managing and living as well as possible with their pain. While understandable, some patients with FM exert considerable effort into resisting their pain experience, including both the physical sensation of pain as well as thoughts and emotions about the impact of pain. Paradoxically, pain resistance strategies amplify physical and emotional pain in the long run and become an increasingly predominant focus of attention and energy that can distract from quality of life improving coping. For example, patients with FM may reduce physical activity, withdraw from social activities, and avoid thoughts of pain. Other patients with FM may use other maladaptive coping strategies such as ruminating on the causes of the pain, frequently checking for pain sensations, and seeking multiple medications to relieve the pain. Avoidance of discomfort is a natural reaction and can sometimes result in short-term pain reduction; however, in the long-term, avoidance strategies can be counterproductive, reducing pain tolerance, increasing pain severity, and undermining coping self-efficacy. The aim of ACT is to reduce the dominance of pain in a patient’s life by allowing pain to exist while engaging in valued activities, thereby improving functioning and quality of life. To achieve this, ACT for chronic pain teaches several skills: acceptance and mindfulness, observing without reacting to distress, and repeated engagement in values-based activities to improve emotional, social, and physical functioning. While the focus of ACT is not expressly on symptom reduction, symptom reduction typically does occur during treatment, as well as improvements in emotional, social, and physical functioning (Du et al., 2021; Herbert et al., 2022; Lin et al., 2017; Luciano et al., 2014; Morcillo-Munoz et al., 2022; Rickardsson et al., 2021; Simister et al., 2018; Trindade et al., 2021; Wicksell et al., 2013).

Despite the evidence that ACT approaches are beneficial for people with FM, access is often limited due to a lack of trained providers, discouraging referral pathways, distance to treatment centers, low insurance reimbursement, and prohibitive costs (Karekla et al., 2019; Robinson et al., 2012). A prescribed self-guided digital ACT therapeutic for FM would represent a significant step towards making this nonpharmacological approach more widely available to the FM population. In addition, internet- and smartphone-delivered interventions allow patients to engage with therapy when it is convenient for them, access it when added support is most needed, and engage more consistently with therapy for greater potential efficacy (Andersson et al., 2019). Indeed, internet-based digital ACT programs have been shown to effectively decrease anxiety and depression, conditions commonly comorbid with FM (Dahlin et al., 2016; Han & Kim, 2022; Kelson et al., 2019; Lappalainen et al., 2014; Lappalainen et al., 2015; Pots et al., 2016; Rickardsson et al., 2021; Thompson et al., 2021; Tokgoz et al., 2021). Patients’ active participation in treatment and related self-care, including self-accountability between healthcare appointments, is necessary for effective management of FM (Arnold et al., 2012). A self-guided digital ACT therapeutic could provide the means for this patient population to more actively participate in their healthcare and foster increased agency in FM management.

**Fig. 1:**
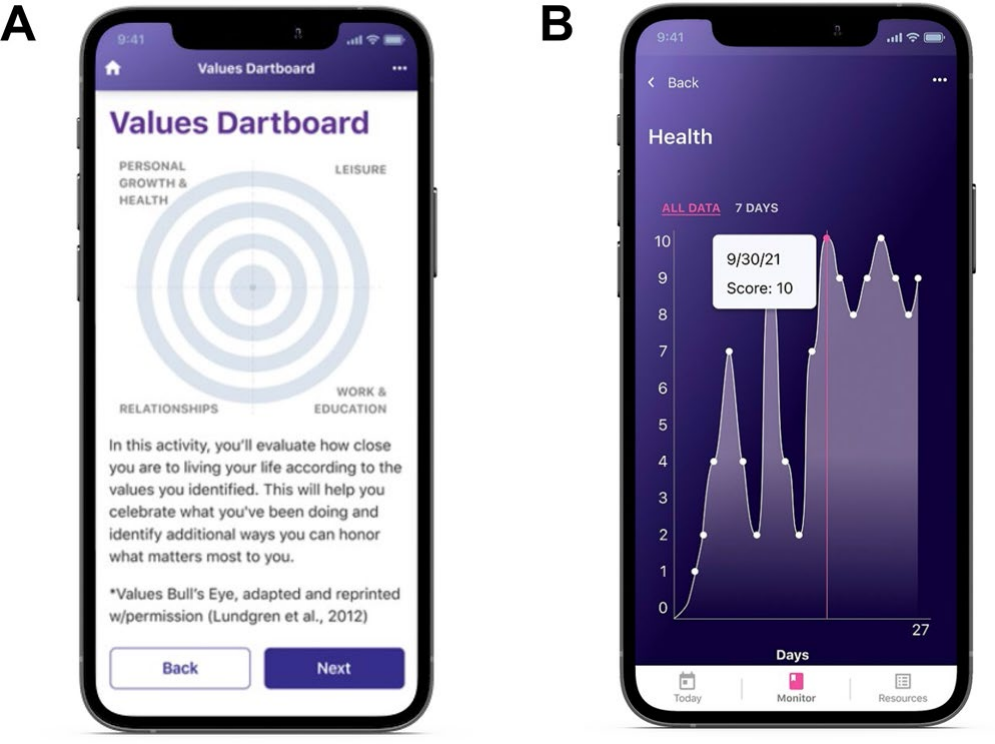
Treatment Application Design. (A) Screenshot of the FM-ACT digital program showing one of the ACT exercises included in the self-guided program. (B) Screenshot of the FM-ST active control showing the daily symptom score monitoring display

To better serve the FM population, we developed a smartphone-based mobile health application (app) that delivers a self-guided, evidence-based ACT program tailored to the management of FM (Stanza, Swing Therapeutics Inc, San Francisco, CA). This investigational digital therapeutic, referred to herein as FM-ACT, was inspired by a web-based ACT program for FM validated by University of Manitoba researchers (Simister et al., 2018) and was recently granted De Novo clearance by the U.S. Food and Drug Administration. The FM-ACT program delivers ACT in 15- to 20-minute daily doses over the course of 12 weeks without the involvement of healthcare providers. The program consists of interactive educational materials that teach ACT skills which are reinforced experientially via values exploration and identification, mindfulness, and relaxation exercises (Fig. 1A). Values-based assignments follow each lesson to assist patients in incorporating ACT skills into their daily lives. Uniquely, FM-ACT teaches additional skills, including self-guided physical exercise and pacing daily activities via a personally customized stepwise, gradual approach.

The SMART-FM pilot study was designed to assess the feasibility of conducting a predominantly virtual study and evaluate preliminary evidence of the safety and efficacy of FM-ACT in preparation for a larger randomized controlled trial. To control for the effects of daily interaction with the app and other study-related biases, an FM symptom tracking app (FM-ST) was developed as an active control. Based on the same platform as FM-ACT, FM-ST enables self-guided daily tracking of patient-reported symptoms and functioning (Fig. 1B). Symptom tracking is commonly used in chronic pain management, and several symptom tracking apps have been developed and validated (Bedson et al., 2019; Ross et al., 2020). FM-ST also provides access to educational materials relevant to FM and general health but does not provide any psychotherapy or healthcare professional involvement. Hence, the FM-ST active control mitigates potential expectation, treatment time and attention, and healthcare provider interaction biases that often occur in chronic pain studies that utilize passive comparison conditions.

A preliminary treatment efficacy evaluation of FM-ACT compared to FM-ST was determined by assessments of improvements in FM severity and impact (primary outcome), patient impression of FM change, depressive symptoms, pain intensity, pain interference, and sleep interference. We hypothesized FM-ACT would improve the above measures more than FM-ST. The feasibility of conducting a digital ACT clinical trial virtually was assessed by evaluating treatment adherence.

## Patients and methods

### Study overview

This 12-week, randomized, multicenter, active-controlled, single-blinded hypothesis, phase II parallel-group study was conducted at 7 independent and experienced clinical research centers in the United States. The study was performed in accordance with the Declaration of Helsinki and in compliance with Good Clinical Practice guidelines, applicable laws and regulatory requirements, and decisions made by the institutional review board (IRB) overseeing the study sites. All participants provided written informed consent. A list of principal investigators, affiliated study sites, and information regarding the overseeing IRB is provided in Supplementary Table 1. In reporting the results of this study, we followed the CONSORT 2010 statement: extension to randomized pilot and feasibility trials (Eldridge et al., 2016). Data were collected from 28 October 2020 to 12 July 2021. The study was retrospectively registered with ClinicalTrials.gov (NCT05005351).

### Investigational device

Stanza is a smartphone-based mobile health intervention that implements an ACT program tailored to the unique presentation of FM (referred to herein as FM-ACT). The 12-week FM-ACT program is composed of 8 core chapters, ideally to be completed in Weeks 1–8, followed by 4 weeks of learned skills practice. Each core chapter is comprised of 4–6 digital therapy sessions in which patients learn the core ACT skills of acceptance, values, mindfulness, defusion, self-as-context, and values-based willingness/committed action. ACT skills are taught via patient education, experiential exercises (e.g., metaphors, visualizations, interactive values identification, etc.), and meditations, and requisite activities are assigned as homework and reviewed at the next session. A descriptive summary of the core program is provided in Supplementary Table 2. In addition to core ACT skills, the FM-ACT program includes other evidence-based interventions for chronic pain including paced physical activities such as household chores (Chapter 3) and paced exercise (Chapter 6). Chapters are unlocked serially; to engage with the next chapter, patients must complete all sessions within the current chapter. Following completion of the core 8 chapters, patients enter the reinforcement portion of the program in which they can select sessions or previously unlocked skills to revisit to suit the individual patient’s needs. The reinforcement sessions are designed to strengthen and engrain the learned skills.

### Study design

The SMART-FM study was designed to (1) assess the feasibility of conducting a largely virtual clinical trial in an FM population, and (2) compare the preliminary evidence for the safety and efficacy of the FM-ACT program to that of the active comparator, FM-ST, for the management of FM. The study included 3 phases: screening and baseline assessment (6–14 days), randomization (1 day), and treatment (12 weeks) (see Supplementary Fig. 1A, Study timeline). After confirmation of study eligibility during the initial in-clinic screening at visit C1, participants were trained in the use of the apps and electronic Patient Reported Outcomes (ePROs) platform. During the screening/baseline assessment period, participants completed daily app practice sessions and a minimum of 2 sets of ePROs spaced 6 − 14 days apart to enable assessment of enrollment eligibility and acquisition of baseline data. After confirmation of study eligibility at visit C2 (remote or in-person), qualified participants were randomized to 12 weeks of treatment with either FM-ACT or FM-ST. Research visits C3-5 were conducted virtually via phone or videoconferencing. The end-of-study appointment (C6/C-ET) was conducted in person or virtually. Because the treatments delivered were inherently impossible to blind, a blinded-hypothesis approach was utilized in which participants were informed they would be assisting in the evaluation of investigational digital interventions potentially helpful in the self-management of FM. All participants were required to complete the core content of the program which consisted of 41 self-guided digital sessions over 12 weeks; program engagement and session completion were tracked electronically. At visits C3-C6, investigators performed safety assessments and monitored protocol adherence. Efficacy assessments were conducted remotely via ePROs during the screening/baseline assessment period, as noted above, and weekly following randomization.

The study was conducted with two successive cohorts. Cohort 1 participants were randomized to FM-ACT or FM-ST (v1) in alignment with the original study protocol. Following a protocol amendment aimed at assessing the effect of symptom tracking and ACT reinforcement on ACT efficacy, Cohort 2 participants were randomized for treatment with a modified version of FM-ST (v2) or FM-ACT with the addition of either daily symptom tracking (FM-ACT + ST) or weekly ACT reinforcement questions (FM-ACT + Insights) (see Supplementary Fig. 1B, Study treatments by cohort). In Cohorts 1 and 2, FM-ST delivered daily app interaction in the form of health education materials and patient-reported symptom and function tracking without delivering psychotherapy. The FM-ST versions differed as follows: the Cohort 1 FM-ST (v1) tracked symptoms in alignment with the Fibromyalgia Impact Questionnaire-Revised (FIQ-R) and pain/sleep interference instruments, while the FM-ST (v2) for Cohort 2 tracked symptoms related to FM impact. FM-ACT for Cohorts 1 and 2 delivered the same core program of 8 weeks of daily 15- to 20-minute ACT lessons followed by 4 weeks of daily reinforcement sessions.

As an unpowered pilot study, formal sample size calculations were not performed. Cohort 1 participants were randomized at a 1:1 ratio into FM-ACT and FM-ST (v1) arms, whereas Cohort 2 participants were randomized at a 1:1:1 ratio into FM-ACT + ST, FM-ACT + Insights, and FM-ST (v2). A dynamic randomization system was used to balance treatment allocations at the site level with a block size of 2 for Cohort 1 and a block size of 3 for Cohort 2.

### Eligibility criteria

Patients 22–75 years of age with a primary diagnosis of FM as defined by the 2016 preliminary ACR FM diagnostic criteria (Wolfe et al., 2016) were eligible for study inclusion provided they had a FIQ-R total score of 25–80, inclusive (Cohort 1) or 35–80, inclusive (Cohort 2), were willing and able to comply with all protocol-specified requirements, were capable of reading and understanding English, and owned and used a smartphone running an appropriate operating system. The FIQ-R eligibility criteria were changed with Cohort 2 to reduce the possibility of a floor effect. Participants were allowed to continue ongoing medications for FM and comorbid conditions provided the dose and regimen were stable for 30 days prior to screening and would remain stable throughout the study period. Permitted concomitant medications included FDA-approved FM medications, non-steroidal anti-inflammatory drugs (NSAIDs), muscle relaxants, anticonvulsants, triptans, ergotamines, serotonin-norepinephrine reuptake inhibitors (SNRIs), and selective serotonin reuptake inhibitors (SSRIs). Medications needed to manage other chronic health conditions, such as diabetes and hypertension, were permitted as well as ongoing nonpharmacological treatments (acupuncture, massage, etc.).

Exclusion criteria included a history of bipolar disorder, current untreated major depressive and/or anxiety disorder, suicidal behavior or ideation in the preceding year, history of significant alcohol and/or drug abuse or dependency, current regular use of systemic corticosteroids, opiates, or benzodiazepines, and medical conditions that could endanger the patient, interfere with the preliminary evaluation of the study device’s safety or efficacy, or compromise the patient’s ability to comply with the study protocol. Patients were also excluded if they were currently undergoing psychotherapy and/or received CBT or ACT for FM or chronic pain in the past 24 months.

Following the screening/baseline assessment period, patients qualified for randomization if they continued to meet the entry criteria, had been compliant with daily app practice sessions, and had successfully completed 2 sets of ePROs 6–14 days apart that resulted in an average baseline FIQ-R total score of 25–80, inclusive (Cohort 1) or 35–80, inclusive (Cohort 2) and an average baseline pain intensity score of 4–9 on an 11-point numeric rating scale (NRS).

### Measures and preliminary efficacy endpoints

#### Fibromyalgia impact questionnaire-revised

The FIQ-R, an updated version of the original FIQ (Burckhardt et al., 1991), is a validated self-report instrument designed to assess the impact of FM on various aspects of a patient’s wellbeing (Bennett et al., 2009). The measure is comprised of 21 questions that address the linked domains of physical function, overall impact, and severity of symptoms. Responses are based on an 11-point NRS and framed in the context of the past 7 days. The FIQ-R total score for each patient ranges from 0 to 100, with higher scores indicating greater disease impact. The FIQ-R was administered electronically immediately following the screening visit (C1), before the baseline visit (6–14 days from completion of the first FIQ-R), and at the end of each week of the treatment period.

Mean change from baseline (CFB) to Week 12 in FIQ-R total scores, analyzed in the intention-to-treat (ITT) population, was utilized as the primary efficacy endpoint. The same assessment analyzed in the per-protocol (PP) population served as a secondary endpoint. A responder analysis of FIQ-R total scores that dichotomized participants into those with ≥ 20% improvement from baseline to Week 12 and those with < 20% improvement, analyzed for the ITT and PP populations, served as a key secondary endpoint. Twenty percent improvement has been utilized in several studies as a threshold of a clinically important difference in FIQ-R scores (Bernardy et al., 2019; Luciano et al., 2014; Luciano et al., 2011).

#### Patient global impression of change

The Patient Global Impression of Change (PGIC) is a PRO version of the Clinical Global Impression scales developed by the U.S. National Institutes of Health. This self-report measure reflects a participant’s belief about the efficacy of treatment and has been validated for studies of psychologically based treatment for chronic pain (Scott & McCracken, 2015). Responses depict a participant’s rating of overall improvement based on a 7-point Likert-type scale. The wording of the assessment used in this study was, “Since the start of the study, overall, my fibromyalgia is:“ with possible responses of “Very Much Improved,“ “Much Improved,“ “Minimally Improved,“ “No Change,“ “Minimally Worse,“ “Much Worse,“ and “Very Much Worse.“

Three analyses of PGIC responses in the ITT and PP populations at Week 12 served as secondary endpoints. The first was a responder analysis that dichotomized participants into those that reported “Very Much Improved,“ or “Much Improved,“ or “Minimally Improved” versus all other responses. A second responder analysis dichotomized participants into those that reported “Very Much Improved” or “Much Improved” versus all other responses. The third analysis treated the PGIC as a continuous outcome with scores ranging from 1 (Very Much Improved) to 7 (Very Much Worse).

#### Pain intensity, pain interference, and sleep interference

Weekly pain intensity, pain interference, and sleep interference scores were self-reported on the same schedule as the FIQ-R using an 11-point NRS. The questions asked were:

1. Pain Intensity: Thinking about the last 7 days, how intense was your pain on average?
2. Pain Interference: Thinking about the last 7 days, how much did your pain interfere with activities at work, leisure, or home?
3. Thinking about the last 7 days, did you have trouble staying asleep because of your pain?

Mean CFB to Week 12 in pain intensity, pain interference, and sleep interference scores, analyzed in the ITT and PP populations, served as secondary efficacy endpoints.

### Safety assessments

#### Adverse events

Adverse events (AEs) and unanticipated adverse device effects (UADEs) were monitored and assessed for clinical significance by study investigators and the Medical Monitor throughout the study. At each visit, participants were queried regarding any AEs that had occurred since the previous visit. For every AE and UADE, the investigator (1) provided an assessment of the severity, causal relationship to study treatment, and seriousness of the event, (2) documented all actions taken, and (3) detailed any other treatment measures taken for the AE.

### Beck depression inventory-II

The Beck Depression Inventory-II (BDI-II) is an extensively validated 21-item self-report inventory of the severity of current depressive symptoms (Beck et al., 1996). Scoring allows for the identification of mild, moderate, and severe levels of depressive symptoms, and for the quantification of change in status over time.

The BDI-II was administered to all participants electronically following the screening visit (C1), prior to the baseline visit (C2), and at Week 12 (C6) or C-ET. At Weeks 4 and 8, Cohort 1 participants were administered the complete BDI-II, whereas Cohort 2 participants only answered BDI-II Question 9 at those time points to assess suicidal ideation.

### Columbia – suicide severity rating scale

The Columbia-Suicide Severity Rating Scale (C-SSRS) is a multiple-item clinician-rated scale that measures suicidal ideation and behavior. Two versions of the C-SSRS were administered by site staff. The Baseline/Screening version was administered at the screening visit (C1) and utilized time frames of “lifetime” and “the past 12 months”. Patients whose responses were indicative of recent suicidal ideation or behavior were excluded from study participation, and appropriate interventions were prescribed. The version administered at the baseline visit (C2) and the final visit (C6 or C-ET) utilized a recall period of “since the last visit.” Patient responses were monitored so that changes indicative of increased suicide risk could be addressed with appropriate actions.

### Statistical analyses

The Safety and ITT populations included all patients who underwent randomization. Participants were excluded from the PP population due to study withdrawal, major medication changes during the study period, failure to complete ≥ 41 app sessions, or failure to meet the amended enrollment criteria (FIQ-R total score ≥ 35). All efficacy assessments were analyzed for the ITT and PP populations. To maximize statistical power, all endpoints were evaluated for the combined FM-ACT group as compared to the combined FM-ST group. ePROs completed by participants prior to the start of treatment were averaged to generate each participant’s baseline scores. When short-term prednisone use was required for acute medical conditions by patients in either arm, participant data was censored from the start of dosing to 7 days after the final dose.

Group demographic and baseline clinical characteristic data were compared using t-tests for continuous outcomes and Cochran-Mantel-Haenszel general association tests for categorical outcomes. For the primary efficacy endpoint and other outcome measures with continuous scoring systems, mean changes from baseline were analyzed using a restricted maximum likelihood (REML)-based mixed model repeated measures (MMRM) approach. The analysis model included the fixed categorical effects of treatment, study time point (week), and treatment-by-week, as well as the continuous covariate of baseline score. Significance tests were based on least-squares (LS) mean values using a predetermined two-sided α of 0.05 and two-sided 95% confidence intervals (CIs). Effect size (Cohen’s *d*) was calculated for continuous endpoints and interpreted as small (*d* = 0.2), medium (*d* = 0.5), and large (*d* = 0.8) in accordance with Cohen and other behavioral therapy studies for FM (Bernardy et al., 2013; Cohen, 1988). For responder analyses, differences in the percentages of responders in the two treatment arms and the corresponding 95% CIs were calculated using the normal approximation; reported *P*-values are based on Pearson Chi-Squared tests (equivalent to difference in proportions z-tests). Summary statistics were used to evaluate treatment adherence.

## Results

### Patient disposition and characteristics

**Fig. 2:**
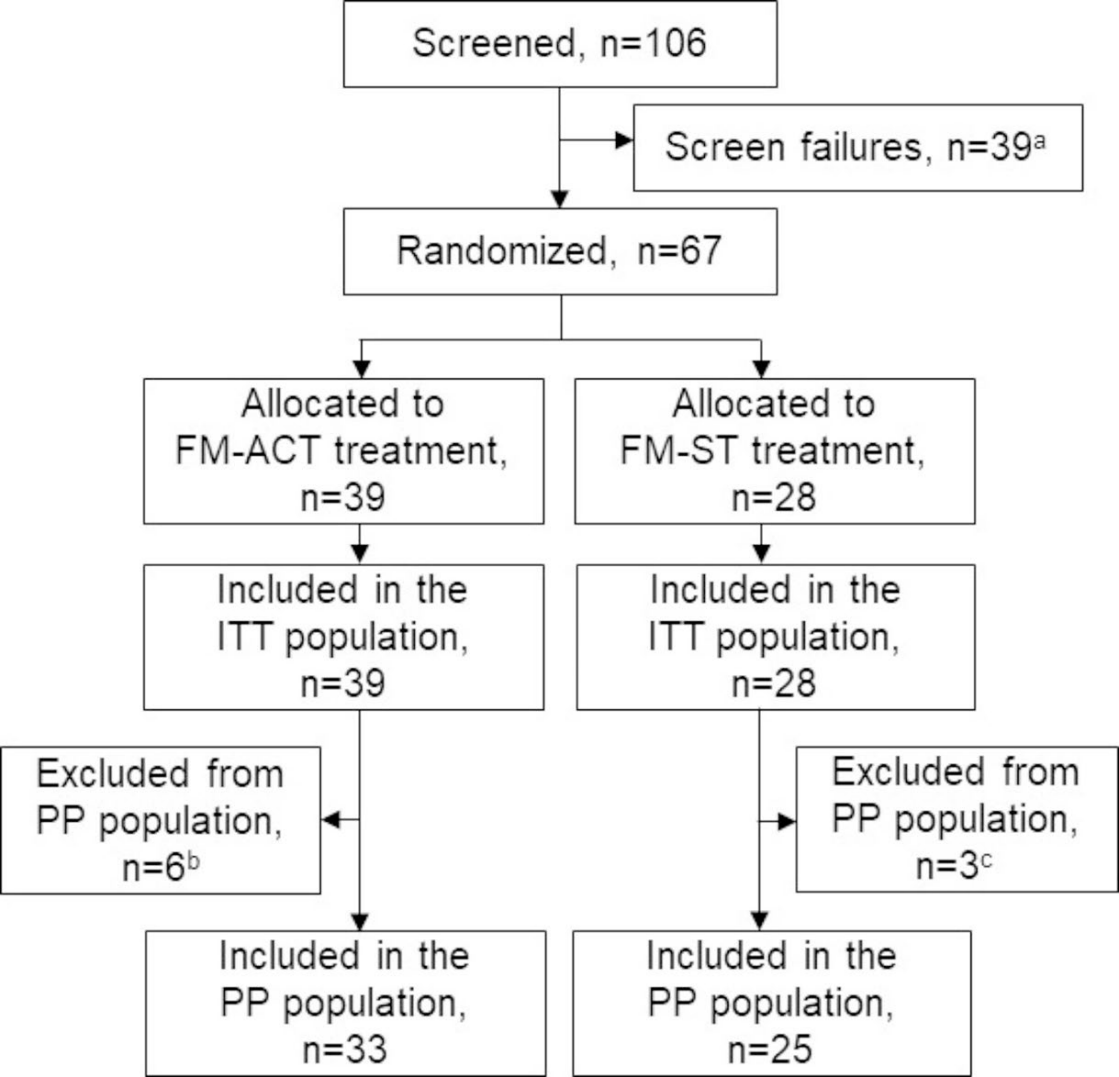
Consolidated Standards of Reporting Trials Flow Diagram Outlining Study Enrollment, Treatment Allocation, and Populations Analyzed. Patients were enrolled from 28 October 2020 to 14 April 2021. The final study assessments occurred on the study completion date of 12 July 2021. ^a^The 39 patients lost to screen failures did not meet the enrollment and/or randomization criteria. ^b^Participants were excluded from the FM-ACT PP population due to study withdrawal (n = 2), failure to complete ≥ 41 FM-ACT sessions (n = 2), unallowed medication changes (n = 1), or failure to meet the amended enrollment criteria (FIQ-R total score ≥ 35) (n = 1). ^c^Participants were excluded from the FM-ST PP population due to study withdrawal (n = 1), or failure to complete ≥ 41 FM-ST sessions (n = 2).

Participant flow through the trial is illustrated in Fig. 2. A total of 106 patients with a diagnosis of primary FM were screened following recruitment via study site internal databases and digital advertising. The numbers of participants screened and randomized to each of the study arms at each site are reported in Supplementary Table 3. Sixty-seven participants were randomized to treatment: 39 to FM-ACT (13 to Cohort 1, 26 to Cohort 2) and 28 to FM-ST (14 to Cohort 1, 14 to Cohort 2). Of the 27 participants in Cohort 1, only one did not meet the Cohort 2 enrollment criteria. Nine participants, including 6 in the FM-ACT arm and 3 in the FM-ST arm, were excluded from the PP population due to study withdrawal, major medication changes, failure to complete ≥ 41 app sessions, or failure to meet the amended enrollment criteria (see Fig. 2). Study attrition was minimal, with withdrawals of 2 participants in the FM-ACT arm and 1 participant in the FM-ST arm.

Demographic characteristics, including gender, race, ethnicity, family status, education, employment status, and reason for unemployment, were well-matched between treatment groups (See Table 1). The study population was 98.5% female with an average age of 53 years (range, 25–68 years). Baseline FM clinical characteristics and prior/concomitant medications and therapies were also well matched between groups with two exceptions: the mean number of years since FM diagnosis was greater in the FM-ST group (*P* = .036), and the number of participants utilizing concomitant gabapentin was greater in the FM-ACT group (*P* = .028) (see Table 2). The mean baseline FM symptom severity score was 8 (range, 5–11).

**Table 1:** Patient demographics by treatment arm: safety population

*Study arm*:		*FM-ACT* *(N = 39)*	*FM-ST* *(N = 28)*	*P-value* ^*1*^
*Demographic Characteristic*:				
**Age (years), mean ± SD**		52.2 ± 10.51	53.6 ± 10.14	.580
**Gender, n (%)**				
	Female	38 (97.4)	28 (100)	.397
	Male	1 (2.6)	0	
**Race, n (%)**				
	American Indian or Alaska Native	0	1 (3.6)	.544
	Asian	1 (2.6)	0	
	Black or African American	1 (2.6)	1 (3.6)	
	Native Hawaiian or Other Pacific Islander	0	0	
	White	37 (94.9)	26 (92.9)	
**Ethnicity**				
	Hispanic or Latino	7 (17.9)	4 (14.3)	.692
	Not Hispanic or Latino	32 (82.1)	24 (85.7)	
**Family Status, n (%)**				
	Divorced	11 (28.2)	6 (21.4)	.711
	Living with partner	1 (2.6)	3 (10.7)	
	Married	18 (46.2)	13 (46.4)	
	Separated	1 (2.6)	0	
	Single	6 (15.4)	5 (17.9)	
	Widowed	2 (5.1)	1 (3.6)	
**Education, n (%)**				
	High school graduate or GED	4 (10.3)	0	.333
	Some college	12 (30.8)	11 (39.3)	
	College graduate	16 (41.0)	13 (46.4)	
	Graduate degree and beyond	7 (17.9)	4 (14.3)	
**Employment status n (%)**				
	Not employed	19 (48.7)	12 (42.9)	.638
	Employed	20 (51.3)	16 (57.1)	
**Reason not employed, n (%)**				
	Seeking employment	2 (10.5)	1 (8.3)	.584
	Homemaker	3 (15.8)	0	
	Retired	8 (42.1)	5 (41.7)	
	Unable to work due to fibromyalgia	3 (15.8)	4 (33.3)	
	Other	3 (15.8)	2 (16.7)	

^1^Between-group *P* values were calculated using t-tests for continuous outcomes and Cochran-Mantel-Haenszel tests for categorical outcomes. GED = Tests of General Educational Development; SD = standard deviation

**Table 2:** Patient baseline clinical characteristics by treatment arm: safety population

*Study arm*:			*FM-ACT* *(N = 39)*	*FM-ST* *(N = 28)*	*P-value* ^*1*^
*Clinical Characteristic*:					
**Years since fibromyalgia diagnosis, mean ± SD**			10.3 (8.14)	15.1 (10.30)	.036
**Widespread Pain Index, mean ± SD**			13.3 (3.46)	13.3 (2.56)	.914
**Symptom Severity Score, mean ± SD**			7.8 (1.34)	8.4 (1.64)	.106
**Fibromyalgia Scale, mean ± SD**			21.1 (3.93)	21.6 (3.01)	.564
**Fatigue (moderate or greater), n (%)**					
		No	3 (7.7%)	1 (3.6%)	.486
		Yes	36 (92.3%)	27 (96.4%)	
**Waking unrefreshed (moderate or greater), n (%)**					
		No	3 (7.7%)	4 (14.3%)	.388
		Yes	36 (92.3%)	24 (85.7%)	
**Cognitive symptoms (moderate or greater), n (%)**					
		No	14 (35.9%)	5 (17.9%)	.109
		Yes	25 (64.1%)	23 (82.1%)	
**Concomitant fibromyalgia medications, n (%)**					
	Duloxetine		8 (20.5%)	4 (14.3%)	.515
	Milnacipran		0	0	
	Pregabalin		1 (2.6%)	3 (10.7%)	.168
	Bupropion		3 (7.7%)	0	.136
	Gabapentin		9 (23.1%)	1 (3.6%)	.028
	SNRIs		2 (5.1%)	1 (3.6%)	.763
	SSRIs		0	1 (3.6%)	.238
	Tricyclic antidepressants		1 (2.6%)	0	.397
	NSAIDs and APAPs		10 (25.6%)	7 (25.0%)	.953
	Muscle relaxants		4 (10.3%)	5 (17.9%)	.372
	Other		2 (5.1%)	2 (7.1%)	.733

^1^Between-group *P* values were calculated using t-tests for continuous outcomes and Cochran-Mantel-Haenszel tests for categorical outcomes

APAP = N-acetyl-para-aminophenol; NSAID = nonsteroidal anti-inflammatory drug; SD = standard deviation; SNRI = serotonin-norepinephrine reuptake inhibitor; SSRI = selective serotonin reuptake inhibitor

### Treatment adherence

A key concern regarding the utility of an at-home, self-guided, smartphone-delivered ACT program was whether participants would use it on a regular basis and complete the prescribed number of daily sessions, particularly in the absence of any involvement from a therapist or other healthcare professional. Treatment adherence was also the primary concern in conducting a predominantly virtual clinical trial with an FM population. Participants in both arms were asked to engage with the treatment a minimum of 5 days per week for 12 weeks, with the core program consisting of 41 sessions. Thus, 41 sessions were prescribed, and 60 were requested. Adherence to treatment was assessed as the total number of sessions completed by a patient during the 12-week treatment period. Session completion was defined as completing all assigned tasks in a daily session. Over the 12-week treatment period, the mean number of sessions completed was 71.00 sessions (SD, ± 16.57) for the FM-ACT arm and 61.89 sessions (SD, ± 13.81) for the FM-ST arm. Thirty-six of the 39 FM-ACT participants (92%) and 26 of the 28 FM-ST participants (93%) completed ≥ 41 sessions over the course of the study.

### Safety

Safety assessments included the evaluation of AEs, including UADEs, increases in depression as assessed by the BDI-II, and changes in the risk of suicide as assessed by the C-SSRS. AEs were monitored and reported throughout the study. There were zero (0) AEs attributed to either treatment. The incidence of AEs during the 12-week treatment period was 25.6% among FM-ACT participants and 17.9% among FM-ST participants. All AEs that occurred over the 12-week treatment period were classified as mild or moderate and included COVID-19 or post-COVID vaccination syndrome (n = 3), anxiety, broken foot, cellulitis, exacerbation of FM pain, exacerbation of gastroparesis, fever, headache, rib contusion, sinus infection, urinary tract infection, vertigo, and worsening anemia (n = 1 each). Safety assessments indicated there was neither increased suicidal ideation nor behavior in either arm during the study.

### Preliminary efficacy

**Fig. 3:**
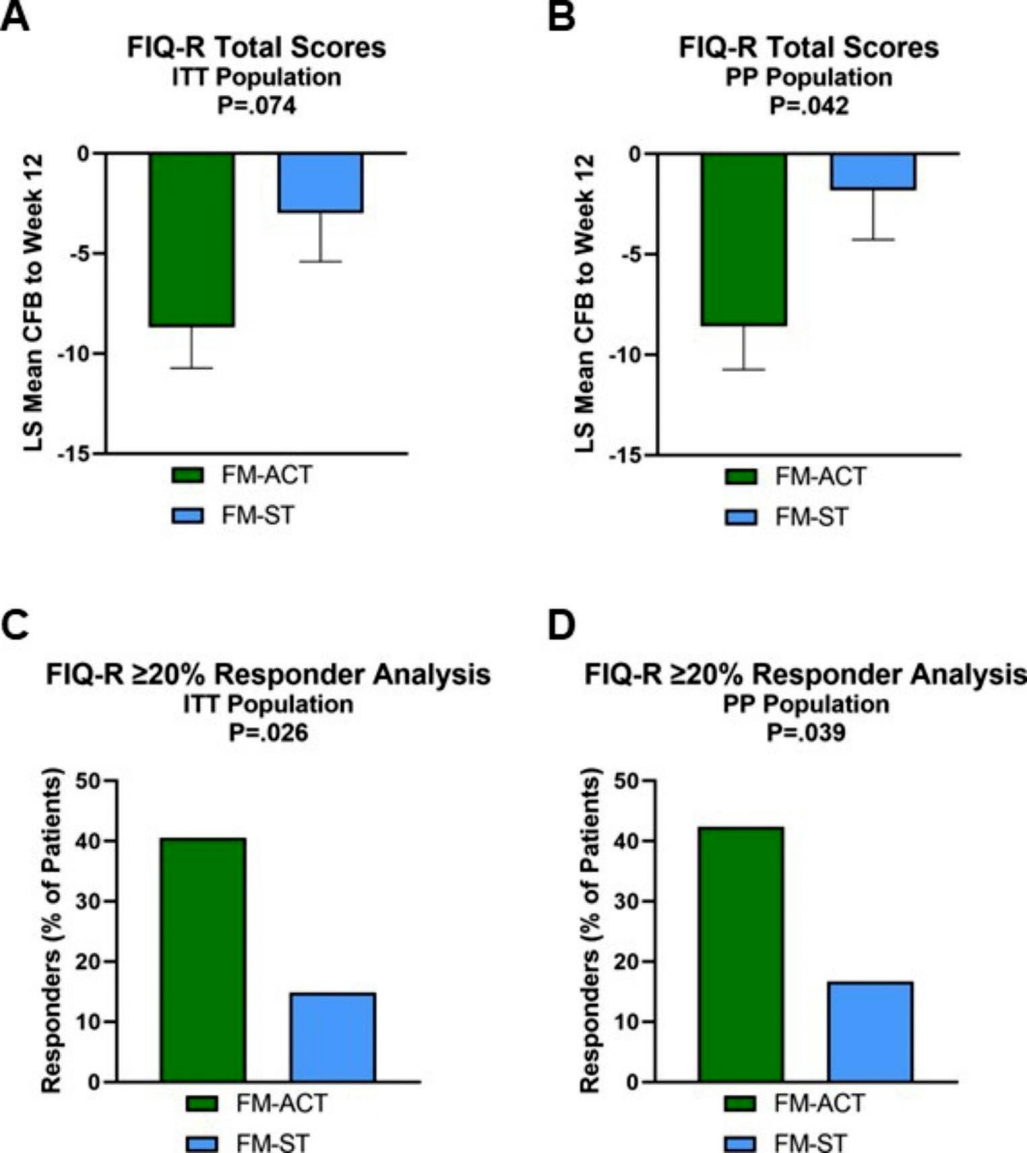
Preliminary Efficacy Analyses Based on FIQ-R Total Scores. The LS mean change from baseline to Week 12 in FIQ-R total scores is shown by treatment arm for the ITT population (A) and the PP population (B). Error bars represent the standard error of the mean. The percentage of participants who reported ≥ 20% improvement from baseline to Week 12 in FIQ-R total scores is shown by treatment arm for the ITT population (C) and the PP population (D)

A summary of preliminary efficacy endpoint analyses is provided in Table 3. The mean CFB to Week 12 in FIQ-R total scores analyzed in the ITT population served as the primary endpoint, and the same assessment analyzed in the PP population served as a secondary endpoint. The between-arm effect size was *d* = 0.44 for the ITT population and *d* = 0.54 for the PP population. Although the ITT population between-arm difference was not statistically significant at the 0.05 level, analysis in the PP population showed a significant improvement in FIQ-R total scores in the FM-ACT arm compared to the FM-ST arm (Fig. 3A-B). FIQ-R total scores were also used for a responder analysis that dichotomized participants into those with a ≥ 20% improvement from baseline to Week 12 and those with < 20% improvement. Although a 14% improvement was determined to represent the minimal clinically important difference (MCID) during validation of the original FIQ instrument (Bennett et al., 2009), an improvement of ≥ 20% has been adopted in several studies as a more stringent threshold for MCID (Bernardy et al., 2019; Luciano et al., 2014; Luciano et al., 2011). In both the ITT and PP populations, treatment with FM-ACT resulted in a significantly greater number of participants who reported a ≥ 20% change in FIQ-R total scores over the 12-week treatment period compared to treatment with the FM-ST active control (Fig. 3C-D).

**Table 3:** Preliminary efficacy analyses for primary and secondary endpoints by treatment arm

	*Study population analyzed*:			*Intention-to-Treat*		*Per-Protocol*	
	*Treatment arm*:			*FM-ACT* *(N = 39)*	*FM-ST* *(N = 28)*	*FM-ACT* *(N = 33)*	*FM-ST* *(N = 25)*
**FIQ-R Total Score**:							
	Mean CFB to Week 12 (SD)			−9.1 (14.71)	−2.2 (12.20)	−8.9 (13.09)	−1.2 (12.65)
	LS mean CFB to Week 12 (SE)			−8.7 (2.03)	−3.0 (2.40)	−8.6 (2.14)	−1.8 (2.47)
	Difference in LS means from ST treatment group (SE)			−5.7 (3.16)	—	−6.8 (3.30)	—
		95% CI for difference in LS means (*P*-value)		−11.9 to 0.6 (*P* = .074)	—	−13.4 to − 0.3 (*P* = .042)	—
		Effect size		0.44	—	0.54	—
**FIQ-R Responder Analysis at Week 12**:							
	≥ 20% CFB in FIQ-R Total Score as responder, n (%)			15 (40.5%)	4 (14.8%)	14 (42.4%)	4 (16.7%)
		Difference in proportions		25.7%	—	25.8%	—
			95% CI for difference in proportions (*P*-value)	5.0–46.5% (*P* = .026)	—	3.2–48.3% (*P* = .039)	—
**PGIC Responder Analysis at Week 12**:							
	Any improvement as responder, n (%)			27 (73.0%)	6 (22.2%)	24 (72.7%)	5 (20.8%)
		Difference in proportions		50.8%	—	51.9%	—
			95% CI for difference in proportions (*P*-value)	29.5–72.0% (*P* < .001)	—	29.6–74.1% (*P* < .001)	—
	Very Much Improved and Much Improved as responder, n (%)			12 (32.4%)	1 (3.7%)	10 (30.3%)	1 (4.2%)
		Difference in proportions		28.7%	—	26.1%	—
			95% CI for difference in proportions (*P*-value)	12.0–45.4% (*P* = .005)	—	8.5–43.7% (*P* = .014)	—
**PGIC Continuous Analysis at Week 12**:							
	Mean at Week 12 (SD)			2.9 (1.08)	4.0 (0.94)	3.0 (1.02)	4.0 (0.95)
	LS Mean at Week 12 (SE)			3.0 (0.16)	4.0 (0.19)	3.0 (0.17)	4.0 (0.20)
	Difference in LS Means (SE)			−1.0 (0.25)	—	−1.0 (0.26)	—
		95% CI for difference in LS means (*P*-value)		−1.5 to − 0.5 (*P* < .001)	—	−1.5 to − 0.5 (*P* < .001)	—
		Effect Size		0.94	—	0.98	—
**FIQ-R Physical Function Domain**:							
	Mean CFB to Week 12 (SD)			−7.6 (16.97)	−4.9 (12.84)	−7.6 (14.01)	−4.2 (13.50)
	LS mean CFB to Week 12 (SE)			−7.4 (2.20)	−5.3 (2.60)	−7.5 (2.25)	−4.4 (2.60)
	Difference in LS means from ST treatment group (SE)			−2.0 (3.41)	—	−3.1 (3.44)	—
		95% CI for difference in LS means (*P*-value)		−8.8 to 4.7 (*P* = .549)	—	−9.9 to 3.8 (*P* = .377)	—
		Effect size		0.15	—	0.23	—
**FIQ-R Overall Impact Domain**:							
	Mean CFB to Week 12 (SD)			−3.2 (4.32)	−0.9 (4.24)	−3.3 (3.81)	−0.8 (4.48)
	LS mean CFB to Week 12 (SE)			−3.0 (0.60)	−1.3 (0.71)	−3.1 (0.62)	−1.0 (0.72)
	Difference in LS means from ST treatment group (SE)			−1.7 (0.93)	—	−2.2 (0.96)	—
		95% CI for difference in LS means (*P*-value)		−3.6 to 0.1 (*P* = .064)	—	−4.0 to − 0.3 (*P* = .026)	—
		Effect size		0.46	—	0.59	—
**FIQ-R Severity of Symptoms Domain**:							
	Mean CFB to Week 12 (SD)			−6.8 (12.75)	0.8 (11.79)	−6.1 (12.52)	1.9 (12.02)
	LS mean CFB to Week 12 (SE)			−6.4 (1.88)	0.1 (2.23)	−5.7 (2.06)	1.1 (2.38)
	Difference in LS means from ST treatment group (SE)			−6.5 (2.93)	—	−6.8 (3.19)	—
		95% CI for difference in LS means (*P*-value)		−12.3 to − 0.7 (*P* = .029)	—	−13.1 to − 0.5 (*P* = .034)	—
		Effect size		0.54	—	0.56	—
**Weekly Pain Intensity**:							
	Mean CFB to Week 12 (SD)			−0.7 (2.16)	−0.2 (1.46)	−0.7 (1.84)	−0.3 (1.50)
	LS mean CFB to Week 12 (SE)			−0.6 (0.27)	−0.3 (0.32)	−0.7 (0.27)	−0.2 (0.31)
	Difference in LS means from ST treatment group (SE)			−0.3 (0.42)	—	−0.5 (0.41)	—
		95% CI for difference in LS means (*P*-value)		−1.2 to 0.5 (*P* = .426)	—	−1.3 to 0.4 (*P* = .265)	—
		Effect size		0.20	—	0.30	—
**Weekly Pain Interference**:							
	Mean CFB to Week 12 (SD)			−1.5 (2.09)	−0.5 (2.02)	−1.5 (1.75)	−0.5 (2.14)
	LS mean CFB to Week 12 (SE)			−1.3 (0.30)	−0.7 (0.36)	−1.4 (0.32)	−0.6 (0.37)
	Difference in LS means from ST treatment group (SE)			−0.5 (0.47)	—	−0.7 (0.49)	—
		95% CI for difference in LS means (*P*-value)		−1.5 to 0.4 (*P* = .256)	—	−1.7 to 0.3 (*P* = .148)	—
		Effect size		0.28	—	0.38	—
**Weekly Sleep Interference**:							
	Mean CFB to Week 12 (SD)			−1.2 (2.28)	0.1 (2.74)	−1.2 (2.09)	0.3 (2.88)
	LS mean CFB to Week 12 (SE)			−1.0 (0.33)	−0.1 (0.39)	−1.1 (0.35)	0.0 (0.40)
	Difference in LS means from ST treatment group (SE)			−0.9 (0.51)	—	−1.2 (0.54)	—
		95% CI for difference in LS means (*P*-value)		−1.9 to 0.1 (*P* = .071)	—	−2.2 to − 0.1 (*P* = .031)	—
		Effect size		0.45	—	0.57	—

CFB = change from baseline; CI = confidence interval; FIQ-R = Fibromyalgia Impact Questionnaire-Revised; LS mean = least-squares mean; PGIC = Patient Global Impression of Change; SD = standard deviation; SE = standard error of the mean

**Fig. 4:**
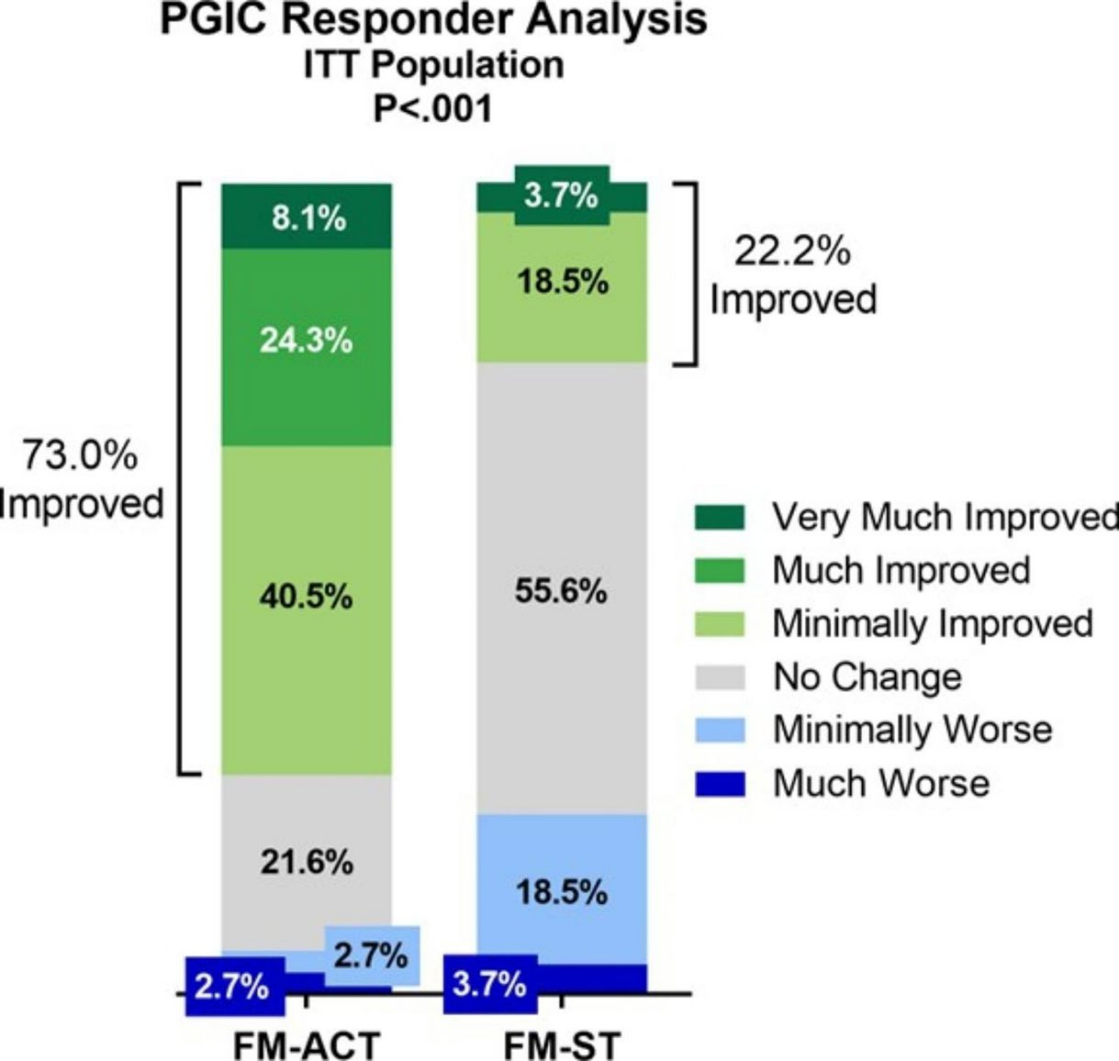
PGIC Responder Analysis at Week 12. The percentages of participants who responded “Very Much Improved”, “Much Improved”, “Minimally Improved”, “No Change”, “Minimally Worse”, or “Much Worse” at Week 12 are shown by treatment arm for the ITT population. No participants reported “Very Much Worse”

Two PGIC responder analyses were performed at Week 12 as secondary endpoints. The first dichotomized participants into those that reported “Much Improved,“ “Very Much Improved,“ or “Minimally Improved” versus all other responses. The second analysis dichotomized participants into those that reported “Very Much Improved” or “Much Improved” versus all other responses. In both analyses, among both the ITT and PP populations, participants who received treatment with FM-ACT reported significantly greater overall global improvement on the PGIC at Week 12 compared to participants who received treatment with the FM-ST active control (See Table 3). Among the ITT population, 73.0% of FM-ACT participants reported improvement at Week 12 compared to 22.2% of FM-ST participants (Fig. 4). The PGIC was also analyzed as a continuous outcome with corresponding scores ranging from 1 (Very Much Improved) to 7 (Very Much Worse). Among both the ITT and PP populations, a large and statistically significant effect size was seen, with FM-ACT participants reporting substantially greater improvement at Week 12 compared to FM-ST participants (*d* = 0.94 and *d* = 0.98, respectively, see Table 3).

Additional secondary endpoints included CFB to Week 12 analyses of scores from the FIQ-R Physical Function, Overall Impact, and Severity of Symptoms domains and the Weekly Pain Interference, Pain Intensity, and Sleep Interference measures. Statistically significant FM-ACT-associated improvements were observed in the PP population for the FIQ-R Impact domain and Weekly Sleep Interference scores and in the ITT and PP populations for FIQ-R Symptoms domain scores (See Table 3).

## Discussion

Although empirically validated in the management of FM, traditional in-person cognitive behavioral therapies, including ACT, are often inaccessible due to a lack of trained providers, physical accessibility, transportation issues, and expense (Karekla et al., 2019; Robinson et al., 2012). The FM-ACT program was designed to make this nonpharmacological approach more widely available to the FM population by providing a completely self-guided ACT program delivered via patients’ smartphones. Results of this multicenter, active-controlled pilot study indicate the FM-ACT program is safe and may be efficacious in aiding FM management. Notably, treatment with FM-ACT resulted in a PGIC between-arm effect size of 0.94, with responder scores superior to those seen with both in-person and internet-based behavioral therapy programs designed for FM (Lumley et al., 2017; Williams et al., 2010). The between-arm effect size for the primary efficacy endpoint of mean CFB to Week 12 in FIQ-R total scores was *d* = 0.44 for the ITT population and *d* = 0.54 for the PP population, favoring FM-ACT. Statistical significance was met for this endpoint in the PP population but not for the ITT population, likely due to the relatively small sample size. In addition, significant improvements with FM-ACT compared to FM-ST that were seen in both the ITT and PP populations included (1) PGIC responder analyses at Week 12; (2) PGIC continuous scoring analysis at Week 12; (3) FIQ-R total scores ≥ 20% CFB to Week 12 responder analysis; and (4) mean CFB to Week 12 FIQ-R Symptoms Domain scores. Significant improvements that were not achieved in the ITT population but were seen in the PP population included (1) mean CFB to Week 12 FIQ-R Impact Domain scores and (2) mean CFB to Week 12 Sleep Interference scores. No device- or study-related safety issues were reported for either treatment arm.

Consistent with the goals and expectations of ACT for patients with FM, the improvements associated with FM-ACT treatment in this preliminary efficacy analysis may have resulted primarily from an increased capacity to adapt and cope, as evidenced by between-arm differences in measures of overall well-being, FM impact, symptoms, and sleep interference. The significant between-arm difference in PGIC scores reflects broad improvement in FM following treatment with FM-ACT. The breadth inherent to the PGIC assessment allows patients to determine, on an individual basis, what a valuable change means to them. Thus, PGIC results are a reflection of what patients perceive as an impactful improvement over the course of the study.

Many of the previously published randomized trials focused on digital behavioral therapies for chronic pain have included psychotherapist-delivered guidance in the form of regular feedback, support, clarification, and/or encouragement (Gandy et al., 2022; Lin et al., 2017; Peters et al., 2017; Rickardsson et al., 2021; Simister et al., 2018; Trompetter et al., 2015). Although guidance and healthcare professional involvement was shown to improve engagement and treatment adherence in one study (Lin et al., 2017), it imposes many of the same limitations to accessibility as traditional in-clinic ACT. Due to lower engagement and higher attrition reported for other digital behavioral therapy programs for chronic pain (Gandy et al., 2022; Herbert et al., 2022; Lin et al., 2017; Trindade et al., 2021; Trompetter et al., 2015), significant effort was expended to make the FM-ACT and FM-ST programs engaging to the patient, including extensive beta testing prior to this study. Engagement with the self-guided ACT program was very high, with FM-ACT participants completing an average of 71 sessions, 31 beyond the prescribed core program. In addition to the high level of engagement observed in both arms, attrition was lower than that seen in other trials examining digital ACT for chronic pain (Herbert et al., 2022; Lin et al., 2017; Morcillo-Munoz et al., 2022; Rickardsson et al., 2021; Simister et al., 2018; Trindade et al., 2021; Trompetter et al., 2015). Hence, the FM-ACT program resulted in an engagement level typically observed with accompanying therapist-delivered guidance while providing the accessibility and scalability of a self-guided platform. Completion of the core treatment by 92% of FM-ACT participants and 93% of FM-ST participants also demonstrates the feasibility of the virtual clinical trial model utilized in the study.

Another distinguishing feature of the current study was the use of an active control. Previously published randomized trials designed to evaluate the efficacy of digital ACT programs for FM or other chronic pain conditions have employed treatment-as-usual (TAU) (Simister et al., 2018) or waitlist controls (Lin et al., 2017; Peters et al., 2017; Rickardsson et al., 2021; Trompetter et al., 2015). In the present study, a self-guided daily digital symptom tracking active control (FM-ST) was developed and utilized to separate the effects of digital ACT from those attributable to daily interaction with the FM-ACT app. This active control was combined with a single-blinded hypothesis approach in which participants were informed they would be assisting in the evaluation of digital interventions believed to be potentially helpful in self-management of FM. This combined approach served to mitigate common potential biases inherent in chronic pain studies, including differences associated with expectation, treatment time and attention, and health care provider interaction. While the use of an active control likely reduced these potential biases, symptom tracking can be effective in FM management and is sometimes considered a treatment in and of itself (Bedson et al., 2019; Ross et al., 2020). Therefore, the use of symptom tracking as an active control may have resulted in fewer between-arm differences compared to other digital ACT for chronic pain trials in which comparisons were made to TAU or waitlist controls.

Study limitations include the small sample size and a homogenous patient population. The small sample (67 randomized participants) likely affected the preliminary efficacy analyses, resulting in nonsignificant between-arm differences for some endpoints. As a pilot study, power calculations were not conducted. However, the results obtained from this study were used in power calculations for a larger, multi-site pivotal trial (ClinicalTrials.gov NCT05243511). The sample size was too small to yield meaningful by-site statistics; therefore, between-site differences were not addressed in the study analyses. Additionally, the study was underpowered to determine whether modifications in the app versions utilized with Cohorts 1 and 2 resulted in meaningful outcome differences. The patient population lacked diversity in race, ethnicity, and gender which limits our ability to demonstrate generalizability. An additional limitation of the current study was the lack of inclusion of ACT process measures as primary or secondary endpoints.

In this pilot study, the self-guided FM-ACT program was beneficial, well tolerated, and provided preliminary evidence of clinically meaningful reductions in the overall impact of FM compared to FM-ST. Patients sustained high treatment engagement and adherence in both arms throughout the study. Numerous studies have shown multimodal approaches provide superior FM outcomes (Carville et al., 2008; Hassett & Gevirtz, 2009; Sarzi-Puttini et al., 2008). The efficacy and engagement observed with FM-ACT treatment, combined with the increased accessibility and scalability of this self-guided ACT therapeutic, suggests the FM-ACT program may provide a promising new component of multimodal FM management. A larger, multicenter study is underway to evaluate further the efficacy of FM-ACT in improving the quality of life for patients with FM.

## Data Availability

Deidentified individual participant study data will be made available to qualified academic investigators for non-commercial research upon reasonable request. Data requests should be directed to the clinical research team at Swing Therapeutics at researchrequests@swingtherapeutics.com.

## Code Availability

FM-ACT and FM-ST application code will not be made available.

## References

[CR1] Andersson G, Titov N, Dear BF, Rozental A, Carlbring P (2019). Internet-delivered psychological treatments: From innovation to implementation. World Psychiatry.

[CR2] Arnold LM, Clauw DJ, Dunegan LJ, Turk DC, FibroCollaborative (2012). A framework for fibromyalgia management for primary care providers. Mayo Clinic Proceedings.

[CR3] Beck AT, Steer RA, Brown GK (1996). Manual for the Beck Depression Inventory-II.

[CR4] Bedson J, Hill J, White D, Chen Y, Wathall S, Dent S, Cooke K, van der Windt D (2019). Development and validation of a pain monitoring app for patients with musculoskeletal conditions (the Keele pain recorder feasibility study). BMC Med Inform Decis Mak.

[CR5] Bennett RM, Friend R, Jones KD, Ward R, Han BK, Ross RL (2009). The revised Fibromyalgia Impact Questionnaire (FIQR): Validation and psychometric properties. Arthritis Res Ther.

[CR6] Bernardy K, Klose P, Busch AJ, Choy EH, Hauser W (2013). Cognitive behavioural therapies for fibromyalgia. Cochrane Database Systematic Review.

[CR7] Bernardy K, Klose P, Welsch P, Hauser W (2019). Efficacy, acceptability and safety of internet-delivered psychological therapies for fibromyalgia syndrome: A systematic review and meta-analysis of randomized controlled trials. European Journal Of Pain.

[CR8] Buckhardt, C. S., Goldenberg, D., Crofford, L., Gerwin, R., Gowens, S., Jackson, K., Kugen, P., McCarberg, W., Rudin, N., Schanberg, L., Taylor, A. G., Taylor, J., & Turk, D. (2005). *Guideline for the management of fibromyalgia syndrome pain in adults and children*. Retrieved from Glenview, IL

[CR9] Burckhardt, C. S., Clark, S. R., & Bennett, R. M. (1991). The fibromyalgia impact questionnaire: development and validation. *J Rheumatol, 18*(5), 728–733. Retrieved from https://www.ncbi.nlm.nih.gov/pubmed/1865419 1865419

[CR10] Carville SF, Arendt-Nielsen L, Bliddal H, Blotman F, Branco JC, Buskila D, Da Silva JA, Danneskiold-Samsoe B, Dincer F, Henriksson C, Henriksson KG, Kosek E, Longley K, McCarthy GM, Perrot S, Puszczewicz M, Sarzi-Puttini P, Silman A, Spath M, Choy EH, Eular (2008). EULAR evidence-based recommendations for the management of fibromyalgia syndrome. Annals Of The Rheumatic Diseases.

[CR11] Clauw DJ (2014). Fibromyalgia: A clinical review. Journal Of The American Medical Association.

[CR13] Cohen J (1988). Statistical power analysis for the behavioral Sciences.

[CR14] Dahlin M, Andersson G, Magnusson K, Johansson T, Sjogren J, Hakansson A, Pettersson M, Kadowaki A, Cuijpers P, Carlbring P (2016). Internet-delivered acceptance-based behaviour therapy for generalized anxiety disorder: A randomized controlled trial. Behaviour Research And Therapy.

[CR15] Du S, Dong J, Jin S, Zhang H, Zhang Y (2021). Acceptance and Commitment Therapy for chronic pain on functioning: A systematic review of randomized controlled trials. Neuroscience And Biobehavioral Reviews.

[CR16] Ehde DM, Dillworth TM, Turner JA (2014). Cognitive-behavioral therapy for individuals with chronic pain: Efficacy, innovations, and directions for research. American Psychologist.

[CR17] Eldridge, S. M., Chan, C. L., Campbell, M. J., Bond, C. M., Hopewell, S., Thabane, L., & Lancaster, G. A. & Group, P. C. (2016). CONSORT 2010 statement: Extension to randomised pilot and feasibility trials. *BMJ, 355*, i5239. 10.1136/bmj.i5239. 10.1136/bmj.i5239 PMC5076380 27777223

[CR18] Fitzcharles MA, Ste-Marie PA, Goldenberg DL, Pereira JX, Abbey S, Choiniere M, Ko G, Moulin DE, Panopalis P, Proulx J, Shir Y (2013). 2012 Canadian Guidelines for the diagnosis and management of fibromyalgia syndrome: Executive summary. Pain Research &amp; Management : The Journal Of The Canadian Pain Society = Journal De La Société Canadienne Pour Le Traitement De La Douleur.

[CR19] Gandy M, Pang ST, Scott AJ, Heriseanu AI, Bisby MA, Dudeney J, Karin E, Titov N, Dear BF (2022). Internet-delivered cognitive and behavioural based interventions for adults with chronic pain: A systematic review and meta-analysis of randomized controlled trials. Pain.

[CR20] Han A, Kim TH (2022). Efficacy of internet-based Acceptance and Commitment Therapy for depressive symptoms, anxiety, stress, psychological distress, and quality of life: Systematic review and Meta-analysis. Journal Of Medical Internet Research.

[CR21] Hassett AL, Gevirtz RN (2009). Nonpharmacologic treatment for fibromyalgia: Patient education, cognitive-behavioral therapy, relaxation techniques, and complementary and alternative medicine. Rheumatic Diseases Clinics Of North America.

[CR22] Hauser, W., Arnold, B., Eich, W., Felde, E., Flugge, C., Henningsen, P., Herrmann, M., Kollner, V., Kuhn, E., Nutzinger, D., Offenbacher, M., Schiltenwolf, M., Sommer, C., Thieme, K., & Kopp, I. (2008). Management of fibromyalgia syndrome–an interdisciplinary evidence-based guideline. *Ger Med Sci, 6*, Doc14. Retrieved from https://www.ncbi.nlm.nih.gov/pubmed/19675740 PMC2703259 19675740

[CR23] Hayes SC, Strosahl KD, Wilson KG (1999). Acceptance and commitment therapy: An experiential approach to behavior change.

[CR24] Herbert MS, Dochat C, Wooldridge JS, Materna K, Blanco BH, Tynan M, Lee MW, Gasperi M, Camodeca A, Harris D, Afari N (2022). Technology-supported Acceptance and Commitment Therapy for chronic health conditions: A systematic review and meta-analysis. Behaviour Research And Therapy.

[CR25] Karekla M, Konstantinou P, Ioannou M, Kareklas I, Gloster AT (2019). The Phenomenon of Treatment Dropout, reasons and moderators in Acceptance and Commitment Therapy and other active treatments: A Meta-Analytic Review. Clinical Psychology in Europe.

[CR26] Kelson J, Rollin A, Ridout B, Campbell A (2019). Internet-delivered Acceptance and commitment therapy for anxiety treatment: Systematic review. Journal Of Medical Internet Research.

[CR27] Kollner V, Hauser W, Klimczyk K, Kuhn-Becker H, Settan M, Weigl M, Bernardy K, Arbeitsgemeinschaft der Wissenschaftlichen Medizinischen Fachgesellschaften. (2012).

[CR28] Lappalainen P, Granlund A, Siltanen S, Ahonen S, Vitikainen M, Tolvanen A, Lappalainen R (2014). ACT internet-based vs face-to-face? A randomized controlled trial of two ways to deliver Acceptance and Commitment Therapy for depressive symptoms: An 18-month follow-up. Behaviour Research And Therapy.

[CR29] Lappalainen P, Langrial S, Oinas-Kukkonen H, Tolvanen A, Lappalainen R (2015). Web-based acceptance and commitment therapy for depressive symptoms with minimal support: A randomized controlled trial. Behavior Modification.

[CR30] Lin J, Paganini S, Sander L, Luking M, Ebert DD, Buhrman M, Andersson G, Baumeister H (2017). An internet-based intervention for Chronic Pain. Dtsch Arztebl Int.

[CR32] Luciano JV, Martinez N, Penarrubia-Maria MT, Fernandez-Vergel R, Garcia-Campayo J, Verduras C, Blanco ME, Jimenez M, Ruiz JM, del Lopez Y, Serrano-Blanco A, FibroQo LSG (2011). Effectiveness of a psychoeducational treatment program implemented in general practice for fibromyalgia patients: A randomized controlled trial. Clinical Journal Of Pain.

[CR31] Luciano JV, Guallar JA, Aguado J, Lopez-Del-Hoyo Y, Olivan B, Magallon R, Alda M, Serrano-Blanco A, Gili M, Garcia-Campayo J (2014). Effectiveness of group acceptance and commitment therapy for fibromyalgia: A 6-month randomized controlled trial (EFFIGACT study). Pain.

[CR33] Lumley MA, Schubiner H, Lockhart NA, Kidwell KM, Harte SE, Clauw DJ, Williams DA (2017). Emotional awareness and expression therapy, cognitive behavioral therapy, and education for fibromyalgia: A cluster-randomized controlled trial. Pain.

[CR34] Macfarlane GJ, Kronisch C, Dean LE, Atzeni F, Hauser W, Fluss E, Choy E, Kosek E, Amris K, Branco J, Dincer F, Leino-Arjas P, Longley K, McCarthy GM, Makri S, Perrot S, Sarzi-Puttini P, Taylor A, Jones GT (2017). EULAR revised recommendations for the management of fibromyalgia. Annals Of The Rheumatic Diseases.

[CR35] McCracken LM, Morley S (2014). The psychological flexibility model: A basis for integration and progress in psychological approaches to chronic pain management. The Journal Of Pain : Official Journal Of The American Pain Society.

[CR36] Morcillo-Munoz Y, Sanchez-Guarnido AJ, Calzon-Fernandez S, Baena-Parejo I (2022). Multimodal Chronic Pain therapy for adults via Smartphone: Randomized Controlled Clinical Trial. Journal Of Medical Internet Research.

[CR37] Peters ML, Smeets E, Feijge M, van Breukelen G, Andersson G, Buhrman M, Linton SJ (2017). Happy despite Pain: A randomized controlled trial of an 8-Week internet-delivered positive psychology intervention for Enhancing Well-being in patients with Chronic Pain. Clinical Journal Of Pain.

[CR38] Pots WT, Fledderus M, Meulenbeek PA, ten Klooster PM, Schreurs KM, Bohlmeijer ET (2016). Acceptance and commitment therapy as a web-based intervention for depressive symptoms: Randomised controlled trial. British Journal Of Psychiatry.

[CR39] Rickardsson J, Gentili C, Holmstrom L, Zetterqvist V, Andersson E, Persson J, Lekander M, Ljotsson B, Wicksell RK (2021). Internet-delivered acceptance and commitment therapy as microlearning for chronic pain: A randomized controlled trial with 1-year follow-up. European Journal Of Pain.

[CR40] Robinson RL, Kroenke K, Mease P, Williams DA, Chen Y, D’Souza D, Wohlreich M, McCarberg B (2012). Burden of illness and treatment patterns for patients with fibromyalgia. Pain Medicine (Malden, Mass.).

[CR41] Ross EL, Jamison RN, Nicholls L, Perry BM, Nolen KD (2020). Clinical integration of a smartphone app for patients with Chronic Pain: Retrospective analysis of predictors of benefits and patient Engagement between Clinic visits. Journal Of Medical Internet Research.

[CR42] Sarzi-Puttini P, Buskila D, Carrabba M, Doria A, Atzeni F (2008). Treatment strategy in fibromyalgia syndrome: Where are we now?. Seminars In Arthritis And Rheumatism.

[CR43] Scott W, McCracken LM (2015). Patients’ impression of change following treatment for chronic pain: Global, specific, a single dimension, or many?. The Journal Of Pain : Official Journal Of The American Pain Society.

[CR44] Simister HD, Tkachuk GA, Shay BL, Vincent N, Pear JJ, Skrabek RQ (2018). Randomized Controlled Trial of Online Acceptance and Commitment Therapy for Fibromyalgia. The Journal Of Pain : Official Journal Of The American Pain Society.

[CR45] Steiner JL, Bogusch L, Bigatti SM (2013). Values-based action in Fibromyalgia: Results from a Randomized Pilot of Acceptance and Commitment Therapy. Health Psychol Res.

[CR46] Thompson EM, Destree L, Albertella L, Fontenelle LF (2021). Internet-based Acceptance and Commitment Therapy: A transdiagnostic systematic review and Meta-analysis for Mental Health Outcomes. Behavior Therapy.

[CR47] Tokgoz P, Hrynyschyn R, Hafner J, Schonfeld S, Dockweiler C (2021). Digital Health Interventions in Prevention, Relapse, and therapy of mild and moderate depression: Scoping review. JMIR Ment Health.

[CR48] Trindade IA, Guiomar R, Carvalho SA, Duarte J, Lapa T, Menezes P, Nogueira MR, Patrao B, Pinto-Gouveia J, Castilho P (2021). Efficacy of online-based Acceptance and Commitment Therapy for Chronic Pain: A systematic review and Meta-analysis. The Journal Of Pain : Official Journal Of The American Pain Society.

[CR49] Trompetter HR, Bohlmeijer ET, Veehof MM, Schreurs KM (2015). Internet-based guided self-help intervention for chronic pain based on Acceptance and Commitment Therapy: A randomized controlled trial. Journal Of Behavioral Medicine.

[CR50] Vincent A, Lahr BD, Wolfe F, Clauw DJ, Whipple MO, Oh TH, Barton DL, Sauver S (2013). Prevalence of fibromyalgia: A population-based study in Olmsted County, Minnesota, utilizing the Rochester Epidemiology Project. Arthritis Care Res (Hoboken).

[CR51] Walitt B, Nahin RL, Katz RS, Bergman MJ, Wolfe F (2015). The prevalence and characteristics of Fibromyalgia in the 2012 National Health interview survey. PLoS One.

[CR52] Wicksell RK, Kemani M, Jensen K, Kosek E, Kadetoff D, Sorjonen K, Ingvar M, Olsson GL (2013). Acceptance and commitment therapy for fibromyalgia: A randomized controlled trial. European Journal Of Pain.

[CR53] Williams DA, Kuper D, Segar M, Mohan N, Sheth M, Clauw DJ (2010). Internet-enhanced management of fibromyalgia: A randomized controlled trial. Pain.

[CR54] Wolfe F, Clauw DJ, Fitzcharles MA, Goldenberg DL, Hauser W, Katz RL, Mease PJ, Russell AS, Russell IJ, Walitt B (2016). 2016 revisions to the 2010/2011 fibromyalgia diagnostic criteria. Seminars In Arthritis And Rheumatism.

